# Chocolate as Carrier to Deliver Bioactive Ingredients: Current Advances and Future Perspectives

**DOI:** 10.3390/foods10092065

**Published:** 2021-09-01

**Authors:** Paulinna Faccinetto-Beltrán, Andrea R. Gómez-Fernández, Arlette Santacruz, Daniel A. Jacobo-Velázquez

**Affiliations:** 1 Tecnologico de Monterrey, Escuela de Ingeniería y Ciencias, Av. General Ramón Corona 2514, Zapopan Jal. C.P. 45201, Mexico; paulinnafb1995@gmail.com (P.F.-B.); andrea.rebeca.gf@gmail.com (A.R.G.-F.); 2 Tecnologico de Monterrey, Escuela de Ingeniería y Ciencias, Av. Eugenio Garza Sada 2501, Monterrey N.L. C.P. 64849, Mexico; asantacruz@tec.mx

**Keywords:** functional chocolate, nutraceuticals, food supplement, carrier of nutraceuticals, delivery system, vitamins, minerals, probiotics, bioactive ingredients, omega-3 polyunsaturated fatty acids

## Abstract

Consumer demand for healthier foods with improved taste and convenience has urged the food industry to develop functional foods added with bioactive ingredients that can supplement basic nutrition (food supplement) or exert a pharmacological effect (nutraceuticals). Chocolate could be used as an ideal carrier to deliver bioactive ingredients, mainly due to its high acceptability by consumers. However, a drawback of using chocolate as functional food is its high sugar content, which impedes its commercialization with the diabetic population. Therefore, there is need to develop sugar-free chocolate formulations added with bioactive ingredients. Nevertheless, sugar replacement and bioactive ingredients addition is a major technological challenge that affects texture, rheology, and sensory properties of chocolate. This review is designed as a practical guide for researchers and food industries to develop the next generation of functional chocolates. Different functional chocolate formulations, including sugar-free, are reviewed as potential carriers for the delivery of bioactive compounds. The physicochemical properties and sensory acceptability of the functional chocolates presented are also highlighted. Finally, future perspectives, such as the use of nanotechnology to improve the bioaccessibility and bioavailability of active ingredients, as well as the need for clinical trials to validate the pharmacological effect of functional chocolates, are also discussed.

## 1. Introduction

Over time, the population’s diet has been adapting due to socio-economic and demographic changes, leading to the development of novel foods with improved taste, nutrition, and convenience. In this context, the food industry has focused on developing foods that can supplement basic nutrition (food supplements) or foods with pharmacological effect (nutraceuticals) that can aid on the prevention and treatment of chronic and degenerative diseases [1,2]. These products are known as functional foods and can be obtained by adding bioactive compounds as ingredients to food formulations [2,3]. The basic principle to design functional foods is inspired by the phrase stated by Hippocrates, the Greek philosopher and father of medicine: “*Allow your food to be your medicine and your medicine to be your food*”. In this context, to develop functional foods, it is of major relevance to identify bioactive ingredients that ensure health benefits either by complementing basic nutrition or by inducing proven health beneficial effects [1]. Examples of these bioactive ingredients include amino acids, proteins, polyphenols, prebiotics, probiotics, omega-3 polyunsaturated fatty acids (ω-3 PUFAs), carotenoids, vitamins, minerals, and plant extracts, among others [2,4].

The addition of bioactive ingredients to foods is a challenge, mainly due to the interactions between the added compound with the food matrix, which generate changes in quality attributes [4]. As a result, the acceptability of functional foods strongly depends on the food matrix selected as carrier of the bioactive ingredients [5]. In this context, chocolate could be used as an adequate vehicle for the delivery of active compounds such as ω-3 PUFAs, probiotics, plant extracts, vitamins, and minerals, among others [1,6,7,8,9,10,11,12,13]. In addition, chocolate formulations naturally contain flavonoids, which are found in cocoa [14,15]. These compounds are highly antioxidant and when administered at adequate amounts have the potential to prevent different chronic and degenerative diseases [15].

A drawback of using chocolate as carrier for the delivery of bioactive ingredients, is its high sugar content in classical formulations. Thus, common chocolates are not suitable for the diabetic population. In this tenor, the demand for sugar-free chocolates has increased over time, urging the food industry and the scientific community to develop sugar-free chocolate formulation through the use of artificial sweeteners (i.e., stevia, erythritol, and isomalt) as sugar replacers [16,17]. However, the development of sugar-free chocolate formulations is a major challenge because the absence of sugar modifies several physicochemical properties related with the acceptability of the product [18,19].

In the present review article, different chocolate formulations added with nutraceuticals are presented as carriers for the delivery of bioactive ingredients. The article presents classical and sugar-free functional chocolates, highlighting the effects of bioactive ingredients addition and/or sugar replacement on the physicochemical properties and sensory acceptability of chocolate. Furthermore, a general protocol that can be used as a guide by the chocolate industry and food scientists, is presented as a tool to develop functional chocolates to be commercialized in the food supplement or nutraceutical markets. Future perspectives such as the use of nanotechnology to improve the bioaccessibility and bioavailability of bioactive ingredients, as well as the need for clinical trials to validate the pharmacological effect of functional chocolates are also discussed.

## 2. Chocolate and Its Natural Flavonoid Composition

Chocolate is generally known as a confectionery product, but humans have consumed it for many years because of its believed health benefits. Indigenous people in Mesoamerica initially consumed chocolate for medical uses as a remedy or a vehicle to deliver medicines. Evidence on the health benefits of chocolate comes from the Kuna Indians in Panama, who consume daily 30 ounces of cocoa base beverage that contains approximately 1880 mg of procyanidins. This tribe presents lower hypertension prevalence and lower rates of diabetes, cancer, and strokes. Therefore, it has been hypothesized that the high intake of procyanidins in this beverage is partially responsible for the lower prevalence of these chronic diseases among the islanders [14]. Once chocolate diffused to Europe, almost 150 uses have been documented for medical treatment, including the improvement of digestion, stimulation of the nervous system, anti-depressant, and improvement of mental performance [14,20].

The health benefits of chocolate are mainly attributed to the antioxidant properties of flavonoids found in cocoa. The cocoa bean is one of the primary sources of dietary phenolics with a 12–18% total dry weight [15]. The main flavonoids found in chocolate cocoa are epicatechin, catechins, and procyanidins; being procyanidins the main contributors to the antioxidant activity of cocoa products [14]. Flavonoids are mainly detected in dark chocolate at approximately the following content per g: 21.5 mg of epicatechin, 10.4 mg catechin, and 821 mg of total procyanidins [14,15]. These compounds have the potential to reduce cardiovascular diseases. Moreover, according with a meta-analysis, cocoa-rich food intake potentially reduces blood pressure [15]. In addition, consumption of cocoa and chocolate have been linked to improve cognitive performance. Animal studies have shown that flavonoids interact directly with molecular targets in the brain generating an antioxidative effect. This effect suggests neuroprotection by improving brain tissue and certain regions involved on memory, learning, and cognition [21].

## 3. Technological Properties to Consider When Developing Functional Chocolate Formulation

Chocolate is one of the most popular confectionary foods due to its pleasant flavor and positive effect on emotions. Chocolate is a complex multiphase system, and thus refining and conching are critical steps to determine the consistency and particle size of the product and reach a specific texture and adequate sensory properties [22]. The milk chocolate system comprises solid particles (cocoa, sugar, and milk powder) dispersed in the fat phase (cocoa butter). The composition of these ingredients affects the final sensory properties and rheological behavior as a fluid mass. To obtain high-quality products, the determination of these properties in chocolate manufacture must be well defined to obtain the right palatable products and fulfill consumers’ preferences [23].

Rheological properties affect the final texture of chocolates, which plays a crucial role in the confectionery industry elaboration process [19]. For instance, if chocolate viscosity is too low, the texture would not be optimal, and if it is too high, bubbles may appear in the molded tablet. In addition to modify texture, viscosity also affects the flavor of chocolate because the taste depends on the order and rate of contact, which is dependent on viscosity and melt rate. Chocolate rheology is usually quantified by yield stress and apparent viscosity parameters. Yield stress provides information related with the transition behavior from elastic to viscous deformation. In other words, it analyzes the change of chocolate from a pseudo-solid to a pseudo-liquid state; this pseudo-liquid state is related to minimum shear stress values. On the other hand, molten chocolate has moisture content mainly from cocoa solids and sugar particles, which may increase friction and apparent viscosity values. Consequently, it is crucial to remove or avoid as much free water as possible to maintain the chocolate’s flow properties and avoid non-desirable microbial growth [24,25].

Furthermore, sensory evaluation is also a key element to evaluate the elaboration process of chocolate and ensure high-quality products that reach consumer’s preferences [26]. Crucial sensory descriptors that determine the acceptability of a chocolate (drivers of liking), include sweetness, melting rate, and sweet aroma; whereas adherence, sandiness, bitterness, and bitter aftertaste are the major drivers of disliking [26].

In general, ingredient composition and processing techniques are essential factors that affect chocolate quality parameters, such as physicochemical properties, rheological behavior, and sensory perception [22].

## 4. Chocolate as Carrier for the Delivery of Bioactive Ingredients

As earlier described, consumers’ demand has led to the development of new products with good taste, health benefits, and conveniences. Thus, confectionary products must adapt to these new challenges through reformulation by adding bioactive components. Chocolate is a suitable matrix, because of its organoleptic characteristics and the protective effect that exerts on bioactive compounds during digestion. One of the benefits of using chocolate as carrier for the delivery of bioactive compounds is that it can mask unpleasant flavors. Therefore, bioactive ingredient such as ω-3 PUFAs, probiotics, phenolic extracts, vitamins, and minerals have been successfully added to chocolate formulations [9,11,12,13,27,28,29,30,31,32,33,34,35,36,37,38,39,40,41]. Likewise, chocolate is also used as an ingredient for different food formulations, thus functional chocolates could also be used to fortify different foods. Furthermore, bioactive ingredients present in functional chocolates could also function as preservative to enhance the shelf-life stability of foods [42]. Results from previous scientific reports on the development of functional chocolate formulations are summarized in Table 1.

### 4.1. Phenolic Compounds

Chocolates added with phenolic compounds have been obtained through the incorporation of plant extracts to formulations (Table 1). Flavonoids are one of the main groups of phenolic compounds present in fruits and vegetables [43]. They can act individually or synergistically with other bioactive compounds to prevent different chronic diseases [44,45]. Flavonoids have been found to regulate protein kinases by the inhibition of transcription factors such as NF-κB which regulates various cytokines and molecules involved in the cellular adhesion associated with inflammation. Consequently, inflammation produces dysregulation of physiological processes, resulting in inflammatory disorders such as cardiovascular, asthma, diabetes, and neurodegenerative diseases or cancer [46]. Therefore, phenolic compounds addition in chocolate in the form of plant extract, in addition to the natural flavonoid content present in cocoa would be highly beneficial to improve human health [33].

Different phenolic extracts from cherry, mulberry, and coffee have been successfully added to chocolate matrices without affecting the sensory acceptability, while increasing their antioxidant activity (Table 1). For instance, it has been found that the addition of cherry fruit (3% *w*/*w*), and coffee waste (2% *w*/*w*) powder in white or dark chocolate matrices increased the total phenol content and DPPH radical scavenging activity [11,34]. Also, the addition of mulberry pomace and peach phenolic extracts to chocolate matrices showed higher flavonoid content and higher antioxidant activity [12,13], the combination of a dark chocolate matrix and encapsulation technologies using chitosan-coated liposomal powders with mulberry phenolic extract, provided better protection of anthocyanins from mulberry and enhanced the in vitro bioaccessability as compared with phenolics added as spray-dried powder [33]. In all these cases, different chocolate formulations added with powders containing phenolic extracts from cherry fruit (3%, *w*/*w*), mulberry pomace (1.5%, *w*/*w*) or coffee waste (2%, *w*/*w*) proved to be a suitable vehicle for the delivery of antioxidant phenolic compounds while preserving the sensory acceptability of chocolate [11,13,34]. On the contrary, dark chocolate added with spray-dried phenolic extracts from peach did not show consumers acceptability due to the bitterness flavor coming from the peach cultivar used [12]. Therefore, it is important to choose the correct source and percentage of phenolic extract added to chocolate formulations to improve the antioxidant effect, while maintaining the acceptability of the product.

### 4.2. Omega-3 Polyunsaturated Fatty Acids (ω-3 PUFAs)

Omega-3 fatty acids (ω3) are polyunsaturated fatty acids (PUFAs) of 18–22 carbons with a double bond at the n-3 position. Mammalian cells cannot synthesize these compounds, and thus they are usually obtained through the human diet. Eicosapentaenoic acid (EPA) and docosahexaenoic acid (DHA) are the two essential ω-3 PUFAs [47]. These fatty acids play a crucial role in maintaining cognitive function in individuals through their neuroprotective properties [47,48]. Populations with a higher intake of DHA present lower risk for developing cognitive decline [49]. Likewise, it has been found that ω-3 PUFAs have a beneficial effect on neuronal function, oxidation, inflammation, and cell death. The ω-3 PUFAs are the major constituents of neuronal membranes, having DHA in a higher proportion (up to 60%). DHA is more rapidly incorporated into gray matter, where neuron cell bodies are located, thus it is essential to develop important brain functions. Therefore, it is important the adequate intake of DHA during childhood to have normal brain function, neurotransmission, vision, and synapsis plasticity [47].

Moreover, several studies suggest that dietary ω-3 PUFAs found in fish oil fight against chronic and degenerative diseases by the inhibition of adipose tissue inflammation. This effect can be reflected in the improvement of insulin sensitivity or reduction of type 2 diabetes incidence [50,51,52]. EPA/DHA anti-inflammatory activity acts on specific receptors and on cell membrane reorganization. Also, EPA helps decrease activation of *cyclooxygenase-1* (*COX-1*) gene, which is involved in inflammation [52]. Furthermore, DHA and EPA improve insulin sensitivity by metabolic pathways such as hepatic lipogenesis, improvement in ketogenesis, reduction of total cholesterol in serum, among others. In addition, ω-3 PUFAs help to increase GLUT4 protein which is involved on glucose transport through plasma membrane [53].

Chocolate formulations have been developed to add ω3 PUFAs using different sources. Milk, white, and dark chocolate have been successfully used as a delivery agent of microencapsulated EPA/DHA from fish oil powder without compromising sensory acceptability [9,35,36]. Likewise, other functional chocolate was developed with the objective to serve as delivery system of ω-3 fish oil and probiotics (*L. plantarum* 299v and *L. rhamnosus*), which could act synergistically to enhance cognitive development in children [30]. The formulation presented optimal results in the rheological, texture, and color analysis, and thus it was concluded that milk chocolate is a suitable vehicle to deliver probiotics and an adequate amount of ω-3 PUFAs (76 mg per 12 g of chocolate). Likewise, the authors stated that if higher levels of ω-3 PUFAs are desirable in the formulation, it would be required to evaluate other sources or presentation of the active compounds (i.e., microalgae oil, microencapsulated fish oil), since fish oil addition at higher concentrations resulted on a product with low sensory acceptability by consumers [30] (Table 1). Additionally, new trends in the nanotechnology field have shown a solution to improve the bioavailability and stability of ω-3 PUFAs. An example is ω-3 PUFAs nano-capsules developed to mask odors and taste of fish oil [54].

### 4.3. Probiotics

The human gut is the site with the highest number of microorganisms in the human body. It has been reported that the intestinal microbiota is involved in various physiological processes, including the activation of the central nervous system (CNS), and there is evidence of communication of the gut–microbiota–brain (GMB) axis [55]. The microorganisms present in the intestine affect the immune system’s regulation; therefore, it can help in the communication of the immune system of the CNS. These could be attributed to the synthesis of neuroactive molecules and metabolites that can modulate the pathogenesis of various neurodegenerative diseases such as Parkinson’s, Alzheimer’s, Multiple Sclerosis, and Amyotrophic Lateral Sclerosis. Intestinal dysbiosis may be behind these diseases by increasing pro-inflammatory cytokines, facilitating the pathogenesis of these disorders. Various studies have revealed that probiotic intake can help intestinal integrity by improving the mentioned disorders [56].

In recent studies, neuroinflammation and oxidative stress have also been associated with an essential role in the pathogenesis of Alzheimer’s influenced by the intestinal microbiota [57]. Therefore, new therapeutic solutions using probiotics have emerged to regulate Alzheimer’s symptoms, such as inflammatory reactions, oxidative stress, β-amyloid plaque deposition, and cognitive functions [58]. Also, probiotics have shown potential to prevent metabolic syndrome and type 2 diabetes. The mechanism of action is through the improvement of lipid profiles by reducing lipopolysaccharide (LPS), low-density lipoprotein (LDL)-cholesterol triglycerides and by decreasing the endoplasmic reticulum stress. In addition, probiotics improve peripheral insulin sensitivity, thus, improving type 2 diabetes [59,60,61]. Furthermore, specific probiotics can promote short chain fatty acids (SCFAs) which maintains secretion of incretin hormone glucagon-like peptide-1 (GLP-1) which inhibits glucagon secretion, improves insulin sensitivity, decreases hepatic gluconeogenesis and enhancer central society [62,63,64].

Different types of chocolates have proven to be an adequate vehicle for the delivery of different probiotic strains (*Lactobacillus* and *Bifidobacterium*) (Table 1). Formulation of a milk chocolate added with freeze-dried probiotic strains (*Lactobacillus acidophilus* NCFM, *Lactobacillus rhamnosus* HN001, and *Bifidobacterium lactis* HN019) proved to maintain 1 × 10^8^ CFU per g of chocolate and >90% of viability after six months of storage at 20 °C [37]. Similar results were reported for the formulation of a dark chocolate added with *Lactobacillus plantarum* showing a final concentration of 6.5 log CFU/g and 81% survival rate after 84 days of storage. In addition, a milk chocolate was developed using two strains of *Lactobacillus* (*Lactobacillus casei* and *Lactobacillus paracasei)* and demonstrated that the use of the chocolate matrix was suitable to maintain bacterial viability between functional level (1 × 10^6^–1 × 10^8^ CFU/g) for 12 months of storage at 18 °C [38].

Moreover, dark chocolate formulation was added with *A. muciniphila* and *L. casei* showing a final bacterial count of >1 × 10^7^ CFU/g after 60 days of storage at 4 °C and 15 °C. Using this chocolate, an in vitro study was performed under gastric transit, where from an initial concentration of 9 log CFU/g the bacterial count was reduced by 1.9 log CFU/g during exposure for 35 min [40]. Similarly, three chocolate formulations (milk, white, and dark) added with immobilized *Lactobacillus casei* 01 and *Lactobacillus acidophilus* LA5 showed to maintain the viability of probiotics > 1 × 10^6^ CFU/g during 60 days of storage at 4 °C. In addition, the three chocolate matrices were appropriate to protect probiotics under in vitro gastrointestinal conditions resulting in 1 × 10^2^ CFU/mL of probiotics at the final stage [41]. Furthermore, the formulation of a milk chocolate matrix using encapsulated probiotics (*Lactobacillus casei* NCDC 298) using sodium alginate (4%) and maize starch (2%) also confirmed that chocolate was a protective matrix to maintain the viability of probiotics. The functionality of this chocolate was tested in an in vivo study, where male albino mice showed an increase in the fecal levels of lactobacilli and a decrease in total coliforms [39]. Finally, a semi-sweet chocolate was added with freeze dried probiotics (*Lactobacillus acidophilus* LA3, and *Bifidobacterium animalis* subsp. *lactis* BLC1) and showed to be a successful matrix maintaining bacterial viability during the chocolate production process (10^8^ CFU/g). In addition, probiotic viability just decreased 1.4 and 0.7 logarithmic cycles during storage (120 days, 25 °C). Finally, formulation of a milk chocolate combined with fish oil and probiotics (*L. plantarum 299v* and *L. rhamnosus GG*) not only maintained ω-3 PUFAs stability, but also maintained the viability of probiotics at 1 × 10^6^ CFU per chocolate portion of 12 g [30]. All chocolate formulations above described, showed that the addition of probiotics to chocolate did not affect the physicochemical properties and sensory acceptability of the final products; demonstrating that chocolate is an excellent food matrix to be used as a vehicle for the delivery of beneficial probiotic bacteria. Furthermore, probiotics in microencapsulated presentation demonstrated higher bacterial viability than when added as freeze-dried powder, thus it would be relevant to perform microencapsulation prior to addition to chocolate formulation. In this context, the use of nanoparticles has shown to be an excellent alternative to improve the viability of probiotics added to chocolates [53]. In addition to the nutraceutical properties of probiotics they also serve for the bio-preservation of food products exerting a dual effect [42].

### 4.4. Vitamins and Minerals

Vitamins and minerals are vital for a proper function of the body and to improve human health. These essential nutrients improve fatigue, cognition, and physiological functions. The inadequate intake of vitamins and minerals vary between countries, because of the different diets and food availability. Therefore, to meet the recommended daily intakes, supplementation of vitamins and minerals is needed [65]. Fortification of foods has gained relevance in the last year, since it has been demonstrated that low blood levels of vitamins C and D, zinc, and selenium correlates with the development of severe cases of COVID-19 disease [66]. Also, the deficiency of minerals such as iron is a frequent nutritional problem around with a significant impact on growth as well as on intellectual and psychomotor development in children.

Food fortification with iron is a practical strategy to prevent iron deficiency. For instance, iron has been added to cookies, candies, brownies, and instant drinks. Likewise, milk chocolate added with iron has been used as vehicle for the delivery of this mineral, while maintaining rheological and sensory properties of the product (Table 1) [28,29]. Moreover, fortification of dark chocolate with vitamin D_3_, has been achieved, obtaining a formulation with no significant changes in the sensory, rheological, melting, and color properties of the final product. Furthermore, when vitamin D_3_ was added in liposomes, the retention of the vitamin was improved during storage (15, 30, 45, and 60 days at 25 °C) as compared to vitamin D_3_ added as free compound (Table 1). Therefore, there is evidence that chocolate could be used as an effective carrier for the supplementation of vitamins and minerals. It is also important to consider that dark chocolate is a natural good source of essential elements, such as potassium and phosphorus [67].

## 5. Sugar-Free Chocolates as Carrier for the Delivery of Bioactive Ingredients

While the use of sucrose as an ingredient prevails in the traditional chocolate industry, numerous low-caloric and non-caloric sweeteners offer new manufacturing opportunities. As mentioned before, sucrose in chocolate manufacture is essential due to its functionality as a bulking agent, texture modifier, mouthfeel modifier, flavor enhancer, and as a preservative [68,69,70]. However, high sugar content of chocolate led to the search for low-calorie and healthier sugar-free alternatives.

Several sugar-free chocolate formulations added with bioactive compounds have been previously reported in literature and are summarized in Table 2. Some of the typical sweeteners used are erythritol, stevia, polydextrose, and inulin. Sugar-free chocolate formulations added with nutraceuticals developed until now contain probiotics, herbs/fruits extracts, and peptides. For instance, inulin with different degrees of polymerization (DP <10 and DP >23) were added as sugar replacer to a white chocolate formulation containing probiotics (4 × 10^8^ CFU/g of *Lactobacillus paracasei* Lpc-37 and 2 × 10^8^ CFU/g of *L. acidophilus* La-14). Results showed that the sugar-free white chocolate formulation was suitable to maintain probiotic viability at 4 × 10^6^ CFU/g after storage (90 days, 20 °C), and showed adequate acceptability by consumers [8]. Similarly, dark chocolates were formulated using probiotics (*Lactobacillus paracasei* and *Lactobacillus acidophilus)* in different formulations of inulin (9% *w*/*w* DP > 23 or 9%*w*/*w* DP < 10) and sucrose (32.5% *w*/*w*), or inulin (9% *w*/*w* DP >23 or 9% *w*/*w* DP < 10) and maltitol (32.5% *w*/*w*). Results showed that sugar-free chocolate samples maintained the viability of probiotics at 1 × 10^6^ CFU/25 g after 90 days of storage. Inulin DP < 10 was more suitable for chocolate production adding probiotics and did not affect physicochemical properties. Quality parameters (color, melting properties, and texture) changed slightly compared with the sucrose control samples [69]. Likewise, Gómez-Fernández et al. [31] evaluated sugar replacement in dark chocolate formulation added with microencapsulated probiotics, considering different sweeteners options (inulin, isomalt, stevia, and polydextrose). Probiotics added were *Lactobacillus plantarum* 299v (L.p299v) and *Lactobacillus acidophilus* La3 20 (DSMZ 17742) at a ratio of 1 × 10^13^ CFU/g which have potential for the treatment of diabetes [31]. The viability of probiotic was maintained at 1 × 10^6^ CFU per chocolate portion (12 g) after the production process, while the formulation with the highest acceptability contained isomalt and stevia as sugar replacers. This formulation has potential to be commercialized as a functional food for the diabetic population [31].

Similarly, a sugar-free milk chocolate formulation added with microencapsulated probiotics and fish oil rich in EPA/DHA was developed, using isomalt and stevia as sugar replacers (Table 2) [32]. The authors observed that the sugar-free milk chocolate matrix added with probiotic and fish oil was capable to maintain probiotic viability (>2 × 10^7^ CFU per portion of 12 g) as well as the concentration of PUFAs (>130 mg per portion of 12 g) [32]. On the other hand, other authors [16] developed a sugar-free dark chocolate functional formulation, using erythritol, stevia, and isomalt as sugar replacers and extracts from beetroot, jamun seed, and pink pitahaya as bioactive ingredients, as well as coffee to mask the aftertaste. Beetroot powder addition resulted in an increase in protein and fiber content in chocolate [16]. Finally, sugar-free functional dark chocolate was developed using isomalt, inulin, and maltitol as sweeteners, and added with αs1-casein peptide as functional ingredient to alleviate stress [71]. The nutraceutical property o the chocolate formulation was proven through a double-blind randomized controlled trial. All the studies above-described show that sugar substitution with sweeteners and the addition of bioactive compounds under certain concentrations, could result in innovative chocolate formulations with similar rheological and sensory parameters as compared with traditional chocolates. Thus, sugar-free chocolates added with nutraceuticals have a high potential to be introduced as a next-generation of functional foods that can effectively be used as carrier for the delivery bioactive ingredients.

There is a need to continue developing functional sugar-free chocolates that could be used as a vehicle for the delivery of bioactive compounds. Therefore, in the present article, the main findings on physicochemical properties and sensory acceptability of sugar-free formulations previously reported in the literature are summarized in Table 3 [73,74,75,76,77,78,79,80]. All those formulations showed adequate rheological properties and acceptability as compared with traditional chocolates. For instance, milk chocolate added with inulin (15% *w*/*w*) or with maltitol (34% *w*/*w*) and inulin (9% *w*/*w*) showed similar physicochemical characteristics as compared with the control [73,77]. Furthermore, the use of isomalt (38% *w*/*w*) as sugar replacer, showed better results for apparent viscosity and yield stress (presenting a solid-like behavior) as compared with inulin (38% *w*/*w*) and maltitol (38% *w*/*w*) formulations [78]. In addition, sugar-free dark chocolate formulations containing inulin (12% *w*/*w*) and polydextrose (36% *w*/*w*) or polydextrose (75% *w*/*w*) and inulin (24% *w*/*w*) showed increased hardness and improved rheological properties [68,74]. Other formulations of sugar-free milk chocolates using polydextrose (60% *w*/*w*) and lactitol (40% *w*/*w*) as sugar replacers presented acceptable and similar sensory characteristics as compared with the control [75]. Similarly, milk chocolate formulated with polydextrose (17% *w*/*w*), erythritol (26% *w*/*w*), and sucralose (0.06% *w*/*w*), showed better quality characteristics as compared with chocolates using stevia and sucralose [76]. This could be attributed to the increase on bitter flavor generated by stevia addition [79]. The information presented in (Table 3) could be used as starting point to further evaluate the addition of compounds with different bioactivities, that could function synergistically for the prevention and treatment of chronic diseases. In this context, a practical guide previously published in literature could be used to design effective nutraceutical combinations and use chocolate as vehicle for their delivery [2]. Moreover, it is important to recall that chocolate, regardless of the addition of sugar or sweeteners, contains antioxidant phenolic compounds that can prevent different chronic diseases, thus formulations shown in (Table 3) may also provide beneficial effect to human health.

## 6. The Chocolate Making Process: Where to Add the Bioactive Ingredients?

**Figure 1:**
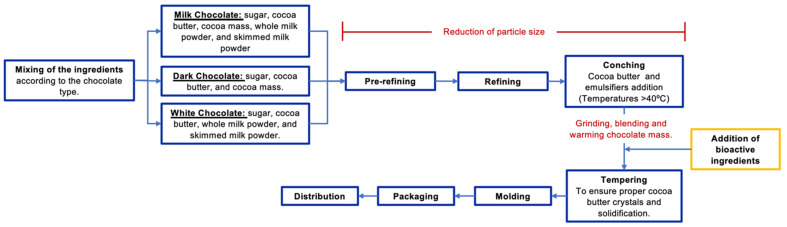
General bioprocess to obtain functional chocolates added with bioactive ingredients. Bioactive ingredients are added at the beginning of the tempering process to minimize degradation of chemical compounds induced by temperature during conching, and to preserve the viability of probiotic bacteria added.

To produce functional chocolates, it is important to adapt the general chocolate making process and select a proper step to add the bioactive ingredients. Factors to consider are the stability of the bioactive molecules to processing conditions (i.e., temperature) and the homogeneous distribution of the bioactive compound in the final product. The general procedure to obtain different types of functional chocolates is shown in Figure 1. The process starts by mixing chocolate liquor/cacao and cocoa butter with sugar and other ingredients, such as milk. Thereafter, the chocolate mix is subjected to pre-refining, refining, and conching, where cocoa butter and emulsifier ingredients are added [70]. Refining and conching are two crucial steps since both determine the particle size, which affects physicochemical properties that could affect consumers’ acceptability [8]. The aim of refining is to reduce the particle size from 100–150 μm to 15–20 μm, since particle size higher than 20 μm are detected by the human tongue [24].

Conching is a critical step, where the inactivation of micro-organisms occurs and is directly associated with the development of quality characteristics such as color, texture, aroma, and flow behavior [81]. In general, this process consists of mixing, shearing, and aeration of the chocolate mass during heating at a specific temperature (>40 °C) [81]. Once the chocolate mass is conched, the next step is tempering; this is another critical step in chocolate’s physicochemical characteristics since this step aims to obtain a solidified chocolate bar. Therefore, it is needed to ensure proper cocoa butter crystals such as β V [82]. These β V crystals can be obtained through temperature and depend on the fat phase’s nature in the chocolate [70]. At the beginning of the tempering process, bioactive ingredients are added into the chocolate matrix to maintain their chemical stability or their viability in the case of probiotic bacteria. Bioactive ingredients are not added during conching because the high temperature affects the stability and/or viability of the bioactive compounds or probiotic bacteria added [9,30,35,36]. After bioactive ingredients addition and tempering, the chocolate is ready for molding, packaging, and distribution for sale (Figure 1).

## 7. General Protocol to Develop Functional Chocolates to Be Commercialized in the Food Supplement of Nutraceutical Markets

**Figure 2:**
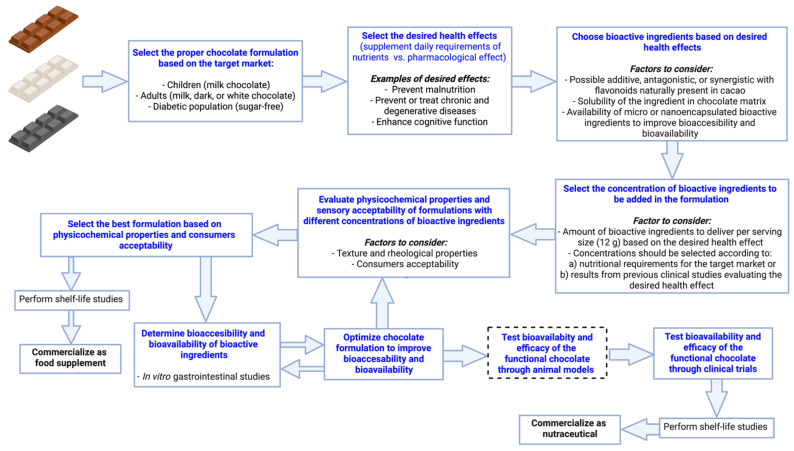
Proposed general protocol to develop functional chocolates to be commercialized in the food supplement or nutraceutical markets. The step marked with dashed lines could be avoided depending on the bioactive compounds selected and their concentrations in the formulations. The in vivo studies with animal models and the clinical trials need previous approval by an ethical committee.

As described in the previous sections of this review article, developing functional chocolates involve several steps to ensure that the final product reach the desired characteristics. Figure 2 shows a proposed general protocol to develop functional chocolates to be commercialized either as a food supplement or as a nutraceutical. First, the chocolate type (dark, milk, or white) should be selected based on the target market. For instance, if the chocolate is intended for children, a milk chocolate formulation would be adequate, whereas if the chocolate is to be consume by the diabetic population a sugar-free formulation should be considered. After selecting the chocolate type, the desired health effect should be determined to choose the adequate bioactive ingredients to incorporate in the formulation. For example, if malnutrition should be prevented or overcome in a certain population, vitamins and minerals showing deficiencies in children should be selected. Moreover, if the desired pharmacological effect is the enhancement of cognitive function, bioactive ingredients such as ω-3 PUFAs could be incorporated. At this point, it is highly relevant to consider the possible additive, antagonistic, or synergistic effect that flavonoids naturally present in cocoa could exert on the desired pharmacological effect when mixed with the selected bioactive ingredients. If this potential interaction is not desirable, it is recommended to opt for a chocolate type with low flavonoid content, such as white chocolate. Furthermore, when searching for the sources of the bioactive compounds it is important to search for ingredients that contain the compounds either as micro or nonencapsulated form to enhance the bioaccesibility.

Once the chocolate type, desired health effect, and bioactive ingredients are chosen, the next step is to determine the concentrations at which bioactive ingredients should be added to provide enough in one serving size of chocolate (12 g). These concentrations must be defined based on previous in vivo studies or clinical trials. If the objective is to design a functional chocolate to be commercialized in the food supplement market, the concentration of bioactive ingredients should be selected according to the nutritional requirements for the target market selected (children, adult, or the elderly). On the other hand, if chocolate is intended to be commercialized as a nutraceutical, concentration of bioactive ingredients should be selected based on results from previous clinical studies evaluating the pharmacological effect of the selected compounds. After the selection of bioactive ingredient concentrations, different chocolate formulations (i.e., varying bioactive ingredient sources and/or concentrations) should be produced in sufficient amounts to evaluate their physicochemical and sensory acceptability. Also, sensory studies with trained panels evaluating the drivers of liking (sweetness, melting rate, and sweet aroma) and disliking (adherence, sandiness, bitterness, and bitter aftertaste) could be evaluated. The results obtained at this step (from physicochemical and sensory evaluations) would be the basis to select the best chocolate formulation. If the functional chocolate is to be commercialized as a food supplement, a shelf-life study would be required. Chocolate is a shelf-stable food matrix, mainly due to the natural antioxidants present in cocoa, which protect the product from oxidation. Therefore, chocolate could be cataloged as a medium or long shelf-life food product. Typical parameters to consider for the determination of the stability of chocolate during storage include oxidative rancidity, fat migration, moisture migration, texture, and graining [24]. Also, since chocolate is, in general, a microbiologically stable food, sensory acceptability during storage would mandate the expiration date, and thus it must be evaluated in shelf-life studies in addition to the physiochemical properties mentioned. Simple methodologies to determine the sensory shelf-life of microbiologically stable foods, such as the survival analysis, could be followed to stablish the expiration date of chocolate [83]. Moreover, when referring to functional chocolates, where a bioactive ingredient is added, the stability of the active compound must be determined during the shelf-life, to ensure that the amount stated in labeling will be available at the time of consumption. Once the shelf-life is validated, the product is ready to go to the market.

If the new chocolate formulation is to be commercialized as a nutraceutical, further steps in the product development process are required. First, it is important to determine the bioaccesibility and bioavailability of the bioactive compounds added in the formulation. This step could be performed using in vitro gastrointestinal assays, through enzymatic digestion that simulates different intestinal conditions [84]. If the bioaccesability and bioavailability are not as expected, it would be recommended to search for additional sources of the bioactive compounds, for instance nano and microencapsulated bioactive compounds have shown improved bioaccesibility and bioavailability. This step of optimization would require changes in the formulation; thus, it would be needed an additional validation of the physicochemical properties and acceptability of the product prior to going further in the development process. After obtaining the formulation with adequate physicochemical and sensory properties, and with the bioactive compounds bioaccessible and bioavailable, the next steps are to test the bioavailability and efficacy (pharmacological effect) of the functional chocolate either through animal models or clinical trials. Here, depending on the bioactive compounds selected and concentrations, the pharmacological effect of the new chocolate could be directly tested through clinical trials with a previous approval of an ethical committee. Once the health effects of the chocolate are validated, a shelf-life of the final formulation should be performed and, after all these steps, the chocolate could be commercialized as a nutraceutical.

## 8. Conclusions and Further Research Needs

In the present review, the current advances from different research groups on the development of functional chocolates were summarized. The research in this fascinating area of food science has been driven by the constant increase of the functional and medicated confectionery market, which is estimated to growth at a compound annual growth rate (CAGR) of 3.8% [85]. It can be concluded that although significant advances have been achieved in this research field, still several steps on the product development process are needed prior to launch these innovative products into the market as nutraceuticals. Most chocolate formulations added with bioactive ingredients have been developed following an approach that would allow their commercialization as food supplements but not as nutraceuticals. However, all these formulations could be further investigated to determine the bioaccesibility and bioavailability of the bioactive compounds added, as well as to determine their pharmacological effect either through animal models or clinical studies.

There are few studies on the health benefits of chocolates added with bioactive ingredients. Examples of these studies include clinical trials to determine of effect of chocolate on cognitive function, glucose metabolism [86], cardiovascular diseases [87], and body mass composition [88]. However, the number of clinical trials are scarce and, thus, there is an urgent need to perform clinical trials focused on validating the pharmacological activity of chocolates added with bioactive ingredients, since most clinical trials performed evaluating chocolate until now, have been focused on determining the effects of flavonoids present in dark chocolate.

The general protocol proposed herein to develop functional chocolates could be used by the chocolate industry or food scientists as a practical guide that considers all basic steps required prior to launching a new chocolate formulation into the market either as a food supplement or as a nutraceutical. Further research effort in this area should consider the use of nanotechnology to generate bioactive ingredients with higher bioaccesibility and bioavailability of bioactive compounds. This review demonstrates that chocolate is a quite versatile food matrix, with ideal characteristics to be used as carrier for the delivery of bioactive compounds, either for their commercialization as food supplements or as nutraceuticals.

## Figures and Tables

**Table 1:** Effect of bioactive ingredients addition on the physicochemical and sensory properties of chocolate formulations.

Bioactive Ingredient	Chocolate Type	Concentration of Bioactive Ingredient Added to the Formulation	Main Findings	Reference
Phenolic compounds	White and dark	Phenolic extract from cherry fruit was added as powder at 0, 3, 6 or 9% (*w*/*w*).	Total phenol content and DPPH radical scavenging activity increased as the concentration of the phenolic extract increased. Color, hardness, smell, taste, texture, and overall acceptability was significatively higher when the phenolic extract from peach was added at 3% in the chocolate formulation.	[11]
		Phenolic extract from mulberry pomace powder was added as powder at 0, 0.5, 1.5 or 2.5% (*w*/*w*).	Mulberry addition at 1.5% showed the higher score in sensory acceptability. The higher the % of mulberry powder, the higher was the chocolate’s hardness, phenolic and flavonoid content. Phenolic extract addition resulted on a formulation with higher antioxidant activity.	[13]
	Dark	Phenolic extract from peach (Fantasia cultivar) was added as powder at 0, 5, 10 or 15% (*w*/*w*).	Phenolic extract addition (15%) showed a significant increase in flavonoid content and reducing power. Sensory test did not show favorable results (score < 6), because of the bitterness of the peach cultivar.	[12]
		Black mulberry extract (BME) spray-dried powder (0.05% *w*/*v*) encapsulated in chitosan-coated liposomes, was added to natural (pH 4.5) and alkalized cocoa liquors (pH 6 and 7.5) during the last hour of conching at temperatures of 40, 60, and 80 °C, respectively.	Chitosan-coated liposomal powders provided better protection of anthocyanins than spray-dried extract and enhanced in vitro bioaccessability.	[33]
		Phenolic extract from coffee waste was added as powder at 0, 1, 2, 3, and 4% (*w*/*w*).	Total flavonoid content and radical scavenging activity increased as the coffee powder ratio increased in chocolate formulation. Optimal sensory acceptability was observed in chocolate added with coffee extract at 2%.	[34]
ω-3 PUFAs	White	Five chocolate formulations added with ω-3 PUFAs (EPA/DHA) from different sources and presentations were evaluated: control, MAP (5.97 g/100 g chocolate), MAO (2.88 g/100 g), TGO (4.04 g/100 g), and TGM (6.78 g/100 g).	Different ω-3 PUFAs sources affected aftertaste, overall acceptability, and color saturation. Sensory evaluation was within acceptable limits. It was possible to produce functional chocolate added with the different ω-3 PUFAs sources. EPA/DHA addition did not affect melting profiles of chocolate formulations.	[35]
ω-3 PUFAs	Milk	Five chocolate formulations added with ω-3 PUFA (EPA/DHA) from different sources and presentations were evaluated: control, MAP (5.97 g/100 g chocolate), MAO (2.88 g/100 g), TGO (4.04 g/100 g), and TGM (6.78 g/100 g).	Milk chocolate proved to be an effective delivery system of EPA/DHA. Oil from microalgae rich in ω-3 PUFA had a mild impact in rheological parameters. Sligh variation on melting profiles were observed on chocolates added with different sources of ω-3 PUFA. The chocolate formulation with ω-3 PUFAs added as microencapsulated form (TGM) showed the highest acceptability, due to its masking property.	[9]
	Dark	Five chocolate formulations added with ω-3 PUFA (EPA/DHA) from different sources and presentations were evaluated: control, MAP (5.97 g/100 g chocolate), MAO (2.88 g/100 g), TGO (4.04 g/100 g), and TGM (6.78 g/100 g).	Chocolate’s fortification with EPA/DHA was feasible without generating a negative effect on quality. Considering taste and bitterness parameters, microencapsulated source was the closest to the control. Microencapsulated forms did not significantly affect quality and may be a potential source to produce a functional dark chocolate with adequate acceptability.	[36]
ω-3 PUFAs and probiotics	Milk	Each chocolate portion (12 g) contained ω-3 PUFA from fish oil [76.0 ± 5.2 mg or 195.8 ± 6.5 mg] and probiotics mix (*L. plantarum 299v and L. rhamnosus GG*, >1 × 10^6^ CFU).	Probiotics and fish oil addition at 76.0 ± 5.2 mg per chocolate portion showed optimal results in the sensory, rheological, texture, and color analysis. Milk chocolate proved to be a suitable vehicle to deliver probiotics, maintaining 1 × 10^6^ CFU per 12 g and an adequate amount of ω-3 PUFA. If higher concentrations of ω-3 PUFA are needed, alternative sources must be studied.	[30]
Probiotics	Milk	Freeze-dried strains of *Lactobacillus acidophilus* NCFM, *Lactobacillus rhamnosus* HN001, and *Bifidobacterium lactis* HN019 were added to milk chocolate formulation.	Final concentration 1 × 10^8^ CFU per g of chocolate was achieved. Probiotics’ addition increased rheological parameters affecting chocolate flow properties. and its addition at 40 °C improved the sensory properties of chocolate and the survival of *L.acidophilus* and *L.rhamnosus.* Survival of the strains was >90%, six months after storage at 20 °C.	[37]
		Lyophilized probiotics (*Lactobacillus casei* and *Lactobacillus paracasei*) were added at 3.33 g/100 g (*w*/*w*) and 40 °C providing at least 1 × 10^6^–1 × 10^7^ CFU/g.	No significant differences in sensory properties were detected between the treatment and probiotic-free chocolates. Bacterial viability maintained between the functional level of 1 × 10^6^–1 × 10^8^ CFU/g to 12 months of storage at 18 °C. Dark chocolate was an adequate and stable carrier for both *Lactobacillus* strains.	[38]
Probiotics	Milk	Dry mix of skim milk powder, cocoa powder, sodium benzoate, inulin (5% *w*/*w*), *Lactobacillus casei* NCDC 298 free or microencapsulated in sodium alginate (4%), and maize starch (2%) were added to milk chocolate ingredients previously mixed.	The final product (free and microencapsulated probiotics) showed probiotics at 1 × 10^8^ CFU/g during 60 days of storage at 7 °C. Solids, fat, and protein in milk chocolate generated a protective matrix for probiotics. In vivo study using adult male albino mice with encapsulated probiotic chocolate supplementation increased the fecal levels of lactobacilli and decremented total coliforms. Probiotics addition either free or microencapsulated did not affect the sensory quality of chocolate.	[39]
	Dark	Probiotic *Lactobacillus plantarum* isolated from fermented cocoa beans was added at an initial concentration of 1 × 10^8^ CFU/g	Dark chocolate showed to be a suitable carrier for probiotics with an 81.25% survival rate at 84 days of storage at 4 °C; no changes in physicochemical properties were detected due to probiotics addition.	[10]
		The viability of probiotics added as free freeze-dried powder to dark chocolate formulations was evaluated during the shelf-life of the product (60 days at 4 or 15 °C) and under in vitro gastric passage conditions. The probiotic strains used were *Akkermansia muciniphila* and *Lactobacillus casei*.	Dark chocolate conferred efficient protection to *A. muciniphila* (a strict anaerobic strain) and *L. casei* showing a final concentration ≥ 1 × 10^7^ CFU/g of chocolate after 60 days of storage at 4 °C and 15 °C. A high survival rate was observed after in vitro gastric transit at pH 3. Probiotic-added dark chocolate had no significant difference between two commercial chocolates in sensory test.	[40]
	Milk, white, and dark	Three different types of chocolate (white, milk, and dark) were added with immobilized *Lactobacillus casei* 01 and *Lactobacillus acidophilus* LA5. In the tempering step, probiotics powder diluted in skim milk solution (10% *w*/*w*) were added to chocolate. The viability of probiotics was tested under gastrointestinal conditions and during storage at 4 °C or 25 °C for 60 days.	The three types of chocolates were appropriate to protect the viability of probiotics under gastrointestinal conditions (1 × 10^2^ CFU/mL of probiotics at the final stage of the in vitro study) and during storage of the product (>1 × 10^6^ CFU/g of chocolate). Dark chocolate showed higher levels of probiotics than milk or white chocolate, due to its high concentration of cocoa and antioxidant compounds. Also, the addition of probiotics did not affect consumers’ acceptability.	[41]
	Semi-sweet	Formulation of semi-sweet chocolate was added with freeze-dried probiotics (*Lactobacillus acidophilus* LA3 and *Bifidobacterium animalis* subsp. *lactis* BLC1). Probiotics were added during tempering process at a ratio of 1 × 10^10^ CFU/100 g. Chocolate stability and probiotic viability was evaluated under gastrointestinal conditions and during storage (120 days at 25 °C).	Incorporation of freeze-dried probiotics was successful in the chocolate matrix resulting in 1 × 10^8^ CFU/g. The formulation showed bacterial viability with a slight reduction of 1.4 and 0.7 logarithmic cycles during storage for 120 days at 25 °C and under in vitro simulated gastrointestinal conditions. No significant differences were shown in the sensory evaluation between chocolate formulations added with probiotics and the control.	[27]
Minerals	Milk	Chocolate formulation was added with desiccated bovine hemoglobin (DBH) at four different concentrations (0, 4.7, 5.7, and 6.7% *w*/*w*), to obtain 10, 12 and 14% of the required daily intake of iron for children (18 mg) per portion (25 g).	Iron-rich chocolates 4.7% and 5.7% had no significant effect in the overall acceptability as compared with the control. Results showed that it is possible to add 5.7% of DBH with a mixing time of 20 min, obtaining 2.2 mg of iron per portion (12% of the recommended daily intake). The product showed to be microbiologically suitable for consumption, and an alternative functional chocolate to contribute to the increase of iron consumption in children.	[28]
Vitamins	Dark	Formulation of dark chocolates was enriched with vitamin D_3_ in two presentations: free and liposome. Final amount of vitamin D_3_ was 5 µg per 10 g of chocolate.	No significant changes in color, sensory, rheological, and melting properties were detected due to the addition of free vitamin D_3_ and liposome vitamin D_3_. Vitamin retention during 15, 30, 45, and 60 days of storage at 25 °C was higher when added as liposomes than the free compounds. Addition of vitamin D_3_ in liposomes is a successful strategy for the fortification of dark chocolate.	[29]

Abbreviations: EPA, Eicosapentaenoic acid; DHA, docosahexaenoic acid; CFU, coliform forming units; MAP, DHA microalgae powder; MAO, DHA microalgae oil; TGM, microencapsulated powder based on fish gelatin; TGO, fish oil triglycerides; PUFAs: polyunsaturated fatty acids. Milk, white, and dark chocolate formulations differ on the sugar, cocoa liquor, cocoa butter, and full cream milk powder content. Details on the formulation of each chocolate presented can be found on its corresponding reference.

**Table 2:** Effect of bioactive compounds addition on the physicochemical and sensory properties of sugar-free chocolate formulations.

Chocolate Type	Sweetners Added	Concentration of Bioactive Compound Added to the Formulation	Main Findings	Reference
White	Inulin (DP < 10 and DP > 23; 9% *w*/*w*)	Probiotics *Lactobacillus paracasei* Lpc-37 ATCC SD5275 (4 × 10^8^ CFU/g) and *L. acidophilus* La-14 ATCC SD5212 (2 × 10^8^ CFU/g) were added to a sugar-free white chocolate formulation.	Probiotics viability resulted on 4 × 10^6^ CFU/g after storage (90 days at 20 °C). Inulin and probiotics addition affected the quality and rheological parameters, while maintained the acceptability by consumers.	[8]
Milk	Isomalt (32 or 35%, *w*/*w*) and stevia (0.03%, *w*/*w*)	Microencapsulated probiotics ((*Lactobacillus plantarum* 299v (L.p299v) and *Lactobacillus acidophilus* La 3 20 (DSMZ 17742)) were added to chocolate after tempering at 29 °C at a ratio of 1 × 10^13^ CFU/g. Chocolates were formulated adding 790 mg of FO per serving size (12 g), expecting to obtain 200 mg of ω-3 PUFAs.	Probiotic viability showed >2 × 10^7^ CFU per serving size in the final product. Sugar replacement by isomalt and stevia, along with probiotics addition resulted on a chocolate formulation with similar hardness values as compared with the control. The product contained >130 mg of ω-3 PUFAs per portion. Fish oil addition decreased the acceptability of chocolate. However, when fish oil was added in sugar-free formulations, the product showed higher acceptability as compared with the control (with sugar) added with ω-3 PUFAs.	[32]
Dark	Optimum concnetration of erythritol, stevia, and isomalt was determines by response surface methodology (RSM)	Beetroot, Jamun seed, and pink pitahaya powder (4 and 7% *w*/*w*) and coffee (0.79% *w*/*w*) to mask aftertaste.	The formulation optimized by RSM consisted of 21.9 g cocoa butter, 5.1 g erythritol, 0.10 g stevia, and of 14.9 g isomalt per 100 g of chocolate. The overall acceptability of sugar-free chocolates was 8.9. Fortification of sugar-free formulation with beetroot powder resulted in an increase of protein and fiber content as compared with the other extracts added.	[16]
	Mixtures of Polydextrose (26.36%) + Inulin (12% *w*/*w*); or Isomalt (38.32% *w*/*w*) + Stevia (0.03% *w*/*w*),	Microencapsulated probiotics *L. acidophilus* La3 (DSMZ 17742) and *L. plantarum* 299v (L.p299v) were added to a dark chocolate formulation after the tempering step (29 °C) at a ratio of 1 × 10^13^ CFU/g.	Results demonstrated that the chocolate formulation maintained adequate viability of probiotics, resulting on a final product with 1 × 10^6^ CFU per portion of 12 g. The sugar-free dark cholate formulation with the highest acceptability and with nearest physicochemical properties to the control, contained Isomalt+ Stevia + Probiotics.	[31]
Dark	Isomalt, inulin and maltitol (38% *w*/*w*)	Addition of αs1-casein peptide (150 mg) before the conching process.	A clinical trial was performed to determine if daily consumption of to evaluate the effectiveness of daily consumption of 12 g of sugar-free added with 150 mg of αs1-casein peptide in alleviating stress in healthy, normal-weight participants (N = 75). Salivary cortisol was measured as and indicator of stress, along with a self-reportingquestionnaire. Consumption of chocolate added with of αs1-casein peptide reduced stress in the Iranian population. The peptide showed stability during the chocolate elaboration process.	[71]
	Sucrose (32.5% *w*/*w*), inulin (9% *w*/*w* DP > 23 or 9% *w*/*w* DP < 10) and maltitol (32.5% *w*/*w*).	(N = 14) Half of the samples contained sucrose, inulin, and probiotics. The other half contained maltitol, inulin, and probiotics (*Lactobacillus paracasei* and *Lactobacillus acidophilus)*. Physicochemical, probiotic viability, and shelf-life analyses were performed.	Inulin type significantly influenced the viability of probiotics during shelf-life. After 90 days of storage, it was obtained 1 × 10^6^ CFU probiotics per 25 g of chocolate. Inulin DP < 10 was more suitable for probiotic addition and chocolate production. The combination of probiotics and inulin (DP < 10) did not affect the physicochemical properties.	[72]

DP: degree of polymerization, CFU: coliform forming units, FO: fish oil. Sugar-free milk, white, and dark chocolate formulations differ on the content of cocoa liquor, cocoa butter, and full cream milk powder. Details on the formulation of each chocolate presented can be found on its corresponding reference.

**Table 3:** Effect of sugar replacement by sweeteners on the physicochemical and sensory properties of chocolate formulations.

Sweeteners Added	Study Details	Experimental Findings	Reference
Inulin, isomalt, and maltitol	Three milk chocolate samples with inulin (9.0% *w*/*w*) (Control (34% *w*/*w* sucrose), 34% *w*/*w* maltitol, and 34% *w*/*w* isomalt) were tested at different conching temperatures (50, 55 and 60 °C). Physical (color, hardness, and water activity) and rheological properties were examined.	Bulk sweeteners with inulin at different conching temperatures induced changes in the physical and rheological properties of chocolate. It was concluded that maltitol is a suitable substitute in milk chocolates containing inulin since showed closer physicochemical properties to the control as compared with the formulation containing isomalt.	[73]
Inulin and polydextrose	Three dark chocolate samples were tested with different high intense sweeteners (BS used: 12% *w*/*w* inulin and 36% polydextrose), the treatments were control (48% *w*/*w* sucrose without BS), stevia (0.24% *w*/*w*), and thaumatin (0.06% *w*/*w*) extracts. Rheological properties, melting behaviors, and physical properties were tested.	BS increased hardness in chocolates and affected the color parameters. Stevia and thaumatin decreased hardness in chocolates. Sugar-free chocolates showed similar flow and melting properties as the control.	[74]
Polydextrose, lactitol, sucralose, and stevia	Five milk chocolate formulations were produced with different sucrose concentrations (40–52%) to determine sweetness by acceptance test. Then sucrose was replaced by BS (60% *w*/*w* polydextrose and 40% *w*/*w* lactitol) and sweetened with (0.07% *w*/*w*) or stevioside (0.03% *w*/*w*). Physicochemical analyses (moisture, particle size, and viscosity) were performed.	The use of BS in milk chocolates resulted to be acceptable and have similar sensory characteristics to sucrose. Sucralose is the most appropriate sweetener for replacing sucrose in milk chocolate than stevia.	[75]
Neotame, stevia, and sucralose	Four different milk chocolates were tested with a time-intensity method. The samples were control (43% *w*/*w* sucrose) and three different sugar-free chocolates with BS (17% *w*/*w* polydextrose, 26% *w*/*w* erythritol) sweetened with 0.06% *w*/*w* sucralose, 0.22% *w*/*w* stevia and 0.004% *w*/*w* neotame.	The best replacement for sucrose was sucralose meaning to be suitable for diet purposes presenting a similar a time-intensity to the control.	[76]
Stevia, polydextrose, maltodextrin, and inulin	Five milk chocolates containing 0.5% *w*/*w* stevia, 26.8% *w*/*w* polydextrose, 5.0% *w*/*w* maltodextrin and with different types of inulin (15% *w*/*w*, HPX, GR, and HP) were prepared. Physical, rheological, and sensory tests were evaluated.	Sugar-free chocolate with HP inulin obtained similar physicochemical characteristics as compared with the control. According to the sensory analysis, inulin with the highest degree of polymerization (HP) obtained an overall acceptance compared to the control.	[77]
Maltitol, isomalt, and inulin	Four different chocolates formulations were elaborated: control (38% *w*/*w* sucrose), maltitol (38% *w*/*w*), isomalt (38% *w*/*w*), and inulin (38%). Rheological behaviors, as well as chocolate structure, were investigated.	Depending on the molecular structure of BS, the apparent viscosity and yield stress were affected. Inulin and maltitol showed a liquid-like behavior while isomalt and sucrose a solid-like behavior. Temperature influenced the structure network between BS and chocolate matrix.	[78]
Inulin and polydextrose	12 chocolate formulations were evaluated, with different percentages of inulin and polydextrose (0, 25, 50, 75, and 100%). Rheological, microstructure, and physicochemical analysis were performed.	Inulin and polydextrose significantly modify the physicochemical and rheological properties and it depended on their concentration. The optimal concentration was 75% *w*/*w* polydextrose and 24% *w*/*w* inulin to improve rheological properties.	[68]
Maltitol, xylitol, and isomalt	17 milk chocolate formulations were evaluated with different concentrations of maltitol (0, 16, 33, 50, and 66% *w*/*w*), xylitol (0, 16, 33, 50, 66, and 100% *w*/*w*), and isomalt (0, 16, 33, 50, 66, and 100% *w*/*w*) using a ball mill process. Rheological, physicochemical, and sensory analysis were performed.	BS influences physicochemical characteristics. According to their fitted models 11.16% *w*/*w* maltitol, 8.9% *w*/*w* xylitol and 12.93% *w*/*w* isomalt produced the optimum milk chocolate improving chocolate quality parameters. The sensory analysis showed that stevia significantly modifies the taste of milk chocolates by increasing bitterness and generating an undesirable aftertaste; also, polyols lowered the texture acceptability.	[79]
Fructose, sugar alcohols (isomalt and lactitol), inulin, and oligofructose, and natural sweeteners	5 semi-sweet chocolates were evaluated. Chocolate 1 (sucrose), chocolate 2 (fructose, lactitol, stevioside, inulin, oligofructose, and agave syrup), chocolate 3 (fructose, xylitol, oligofructose, liquorice root, yacon, rice syrup, dried carrot, and black locust flowers), chocolate 4 (fructose, isomalt, stevia leaves, oligofructose, lucuma, agave syrup, and peppermint), chocolate 5 (fructose, maltitol, stevia leaves, yacon, and rice syrup).	Chocolate containing inulin, oligofructose, and agave syrup was characterized with the lowest content of sugars but exhibited calorific value the closest to control chocolate, while chocolate containing yacon, carrot, and black locust flowers exhibited the highest sugar content. Polyols increased hardness in the chocolate matrix due to their large particle size.	[80]

BS: Bulk sweetener, HP: High-performance inulin, HPX: High-performance X inulin, GR: Granulated inulin. a Definitions: Time-intensity: Study the substitution of ingredients, especially sucrose, by other sweeteners, in the preparation of a product with equivalent sweetness; provide information on the behavior of the flavor as perceived by consumers during ingestion of the food and is used to obtain the temporal profile of an attribute in a certain product [74]; Ball mill process: used to grind or blend materials by the principle of impact and attrition: size reduction is done by impact as the balls drop from near the top of the shell. Sugar-free milk, white, and dark chocolate formulations differ on the content of cocoa liquor, cocoa butter, and full cream milk powder. Details on the formulation of each chocolate presented can be found on its corresponding reference.

## Data Availability

Not applicable.
